# Demographic Factors Affect the BMAC Proteome

**DOI:** 10.1021/acs.jproteome.5c00804

**Published:** 2026-07-27

**Authors:** Colin Herna, Madison Cipriani, Ranjan Sachdev, Sabrina Jedlicka

**Affiliations:** † 1687 Lehigh University, Bioengineering 5 E. Packer Ave, Bethlehem, Pennsylvania 18015, United States; ‡ Sachdev Orthopaedics, 3735 Nazareth Rd, 302 A, Easton, PA 18045, United States

**Keywords:** statistics, bone marrow aspirate concentrate (BMAC), humeral head, iliac crest, bone marrow, proteome, serum proteomics, smoking, BMAC demographics

## Abstract

Bone marrow aspirate concentrate (BMAC) is a promising orthobiologic therapy, yet the impact of patient demographics on its proteomic profile remains poorly understood. This study investigates how smoking status, age, sex, and BMI modulate BMAC’s protein composition, focusing on 109 analytes across inflammation, immune response, and tissue regeneration pathways. BMAC samples from 120 patients (iliac crest: *n* = 77; humeral head: *n* = 43) were stratified by smoking status (current, former, never-smokers) and analyzed via antibody microarrays. Nonparametric statistical analyses (Kruskal–Wallis, Dunn’s post hoc) with false discovery rate (FDR) corrections revealed significant proteomic alterations in iliac crest-derived BMAC from smokers, including upregulated pro-inflammatory cytokines (IL-17, TNF-α; *p* < 0.05, FDR-adjusted) and downregulated anabolic factors (TGF-β1, BMP-9). Former smokers exhibited intermediate profiles, suggesting partial recovery postcessation. Sex-based differences (e.g., BMP-5, FGF-4) were noted but did not survive FDR correction. Humeral head samples showed minimal smoking-related changes, highlighting site-specific effects. These findings underscore smoking’s catabolic influence on BMAC’s therapeutic potential and advocate for pretreatment cessation protocols.

## Graphical abstract

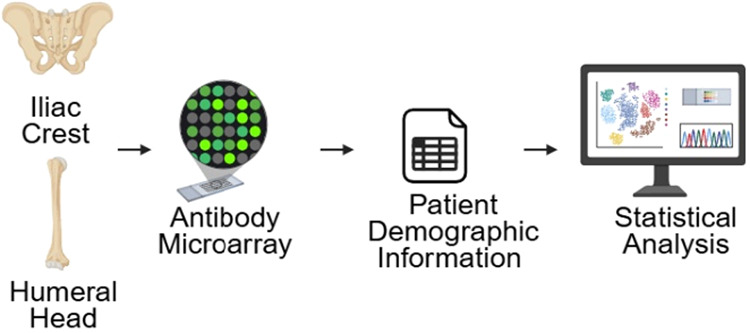

## Introduction

Bone marrow aspirate concentrate (BMAC) has emerged over the last 20 years as a unique treatment within the orthobiologics space. Through a combination of innately present mesenchymal stem cells, anti-inflammatory proteins, and growth factors, BMAC is able to deliver several powerful regenerative agents in a single injectable.^1,2^

To date, the standard for BMAC acquisition involves aspirating bone marrow from the iliac crest region of the hip and concentrating it down through one of many centrifugation methods. To our knowledge the iliac crest has remained as the gold standard harvest site due to its extractable volume of marrow, yield of progenitor cells (MSCs), and its association to preferred patient outcomes.^3–6^

The role of MSCs (Mesenchymal Stem Cells) in bone marrow are well characterized by works examining the osteogenic effects of bone marrow as early as the 19th century. Later bodies of work went on to demonstrate said osteogenic effects could be attributed to a small subpopulation of bone marrow cells.^7,8^ MSCs are formally defined by the Mesenchymal and Tissue Stem Cell Committee of the International Society for Cellular Therapy as, “plastic adherent cells while being maintained under standard culture conditions, they must express CD105, CD73 and CD90, and lack expression of CD45, CD34, CD14 or CD11b, CD79α or CD19 and HLA-DR surface molecules, as well as differentiate to osteoblasts, adipocytes, and chondroblasts in vitro”.^9^ These cells host a variety of remarkable properties which when looked at as a whole, become a sum greater than its parts; Intrinsic tropism more generally thought of as a homing mechanism is the method by which MSCs mobilize, adhere, and infiltrate into an inflammation site.^10^ The anti-inflammatory activity of MSCs then allows for simultaneous upregulation of anti-inflammatory and downregulation of pro-inflammatory cytokines providing both local and systemic relief.^11,12^ MSCs are also capable of apoptotic and immunomodulation through secreted cytokine paired with regulation of B and T lymphocytes, natural killer cells, monocytes, neutrophils, and macrophages.^10,11,13^ Additionally, MSCs are capable of instigating tissue repair and regeneration by way of paracrine signaling, ultimately resulting in regulation of cell proliferation, antioxidant activity, and differentiation.^14,15^ Yet, despite our knowledge about the capacity of MSCs, there are still mixed results insofar as how patient demographic factors such as age, sex, BMI, smoking, and extraction site, affect the resulting concentration of these cells.^4,16–18^

The same ambiguity of patient demographic factors and their effects on BMAC’s MSC concentrations also extend to its proteomic profile. However, the cellular component of BMAC has a more robust body of literature, especially when compared to BMAC’s proteomic counterpart. Extensive proteomic analyses on BMAC are rarely performed despite many studies directly naming proteins such as VEGF, PDGFs, BMPs, IL-1ra, and TGF-β1 as key anti-inflammatory and growth factors housed in BMAC.^2,3,19^ When BMAC proteomic analyses are performed they are typically done through the lenses of the MSC secretome, rather than the native proteins in BMACs “serum” component.^20–23^ The general lack of standardization around the BMAC processing procedure, further propagates the issue of not truly understanding how patient demographic factors modulate both MSC and serum protein concentration.^3,24^

Examples of demographic variability related to BMAC can be seen in the clinical outcomes of recent studies. A 2025 study identified that overweight individuals exhibited inferior functional and radiographic (MOCART) outcomes compared to normal-weight peers following BMAC-supplemented cartilage repairs.^25^ A subsequent 2026 trial sought to use BMAC on hyaluronic acid scaffolds combined with intraarticular PRP full-thickness knee cartilage lesions; this study proactively controlled for BMI and age limiting the treatment to patients with a BMI of 20–30 kg/m^2^ and ages 18–40 to achieve excellent biological healing and structural MOCART 2.0 scores.^26^

Our aim for this work is to evaluate the effects of smoking with respect to patient demographic factors (age, sex, bmi) to better understand how specific proteins may be differentially affected by a patient’s smoking status. There are numerous ways to go about parsing this data, but, given the catabolic nature of smoking in contrast to the anabolic nature of BMAC we have elected to focus on how age, sex, and BMI, interact with smoking.

Consumption of cigarettes typically leads to pathologies like cancer, cardiovascular, and lung disease; According to the CDC (2022) roughly 1 in 5 adults (49.2 million) use cigarettes and these tobacco products are one of the leading causes of preventable disease and death in the United States.^27^ Cigarette smoke itself has been shown to accelerate aging across a variety of tissues in the body,^28,29^ contains reactive oxygen species (ROS) and a myriad of cytotoxic compounds.^30–32^

At the cellular level cigarette smoke exposure (CSE) has been shown to delay chondrogenesis in mice and inhibit osteogenesis in rabbits.^33,34^ Furthermore, in MSC’s derived from adipose tissue, CSE exposure impaired their migration and viability, altered their secreted protein profile, and instigated genetic variations throughout differentiation into osteocyte and chondrocyte lineages.^35^ In vitro bone marrow MSCs were isolated from guinea pigs and exposed to cigarette smoke; authors found that short-term CSE resulted in MSC’s homing, proliferation, differentiation, and migratory properties being diminished.^36^

The damage that smoking causes can be summarized as catabolic in nature, corroding tissues to point of cellular senescence, apoptosis, or proliferative malfunction.

The link between smoking and BMAC as a treatment is not explicitly well documented. Patients are advised to refrain from smoking and alcohol prior to an appointment for BMAC treatment. Currently what we understand is that smoking and BMAC have antagonistic effects in that smoking produces a catabolic response in the body, whereas BMAC produces an anabolic response in the body. For this reason, actively smoking patients are believed to receive reduced to little effectiveness from the BMAC therapy.

## Materials and Methods

### Samples Acquisition

Bone marrow samples were aspirated (BMA) from patients (*n* = 120) presenting symptoms of joint pain (Table 1). BMA was processed into bone marrow aspirate concentrate (BMAC) by Sachdev Orthopedics as part of surgical intervention related to onset of patient pain using a commercially available centrifuge (Magellan, ISTO Biologics) to isolate the buffy coat containing the MSCs. The procedure was performed as a medical operation with informed consent. Leftover materials were deidentified and anonymized prior to being used as part of the subsequent analysis.

**Table 1:** Demographic Characteristics of Patients from Whom Bone Marrow Aspirate Concentrate (BMAC) Samples Were Acquired^a^

bone marrow source	smoking status	age (years)	sex (M | F)	BMI (KG/M^2^)
iliac crest (77)	smoker (15)	56.20 ± 8.03	7 | 8	28.72 ± 3.49
	former smoker (20)	63.20 ± 8.31	10 | 10	31.56 ± 5.16
	never smoker (42)	63.61 ± 10.17	14 | 28	30.84 ± 7.48
humeral head (43)	smoker (9)	58.33 ± 9.86	8 | 1	29.99 ± 7.41
	former smoker (11)	64.45 ± 9.98	6 | 5	30.02 ± 5.47
	never smoker (23)	63.61 ± 10.17	10 | 13	30.77 ± 6.59
all (120)	smoker (24)	57.00 ± 8.61	15 | 9	29.18 ± 5.12
	former smoker (31)	63.65 ± 8.79	16 | 15	30.94 ± 5.24
	never smoker (65)	62.85 ± 11.14	24 | 41	30.81 ± 7.10

a Data are presented for the iliac crest (*n* = 77) and humeral head (*n* = 43) cohorts and further stratified by patient smoking status (smoker, former smoker, or never smoker). The average age, sex, and BMI are reported for each smoking group.

### Ethical Statement on Samples

The specimens in question were collected under a routine clinical procedure, not as part of a clinical trial. The specimens were marked as waste, and further deidentified prior to receipt. As such, Lehigh University did not classify this as Human Subjects Research.

### Sample Preparation

BMAC samples were separated into their serum and cellular components via centrifugation (1500g × 5 min). Each BMAC serum sample was further processed using an Albumin & IgG Depletion SpinTrap (Cytiva) to remove excess solutes, immunoglobulins, and albumin proteins. Once stripped, each BMAC serum sample’s total protein concentration was quantified using a bicinchoninic acid assay (BCA) using the following kit Micro BCA^TM^ Protein Assay Kit (Thermo Scientific, 23227). All kits were used in accordance with their manufacturer protocols. After stripping and determining concentrations of each BMAC serum sample, samples were diluted at a final concentration of 100 μg/mL to standardize the concentration for microarray analysis.

### Microarray Slide Preparation and Scanning

BMAC serum samples at a final concentration to 100 μg/mL were loaded into four antibody microarrays (Table 2) from Ray Biotech; Human immune response array GS1 (GSH-IMR-1–2), Human inflammation response GS3 (GSH-INF-3–2), Custom G-series Select “Osteoclast” Array (GSH-CUST), Custom G-series Select “Cartilage” Array (GSH-CUST). The protocol for processing each set of arrays was provided by Ray Biotech.

**Table 2:** Map of Each Array Used to Characterize the Patient Bone Marrow Aspirate Concentrate Samples Based on 109 Unique Proteins^a^

array protein maps									
human inflammation array GS3					human immune response array GS1				
BLC	Eotaxin	Eotaxin-2	G-CSF	GM-CSF	CD14	CD40	CD163	CRP	E-selectin
I-309	ICAM-1	IFNg	IL-1a	IL-1b	Fas	FAS L	G-CSF	ICAM-1	IL-1a
IL-1ra	IL-2	IL-4	IL-5	IL-6	IL-1b	IL-2	IL-2 Ra	IL-4	IL-6
IL-6sR	IL-7	IL-8	IL-10	IL-11	IL-8	IL-10	IL-12p70	IL-13	IL-18
IL-12p40	IL-12p70	IL-13	IL-15	IL-16	lipocalin-2	MCP-1	MCP-2	MIF	MIP-1a
IL-17	MCP-1	MCSF	MIG	MIP-1a	MIP-1b	OPN	PAI-I	PF4	Procalcitonin
MIP-1b	MIP-1d	PDGF-BB	RANTES	TIMP-1	RAGE	Resistin	ST2	Thrombomodulin	TNFa
TIMP-2	TNFa	TNFb	TNF RI	TNF RII	TREM-1	Troponin I	uPAR	VCAM-1	VEGF
custom G-series select “osteoclast” array					custom G-series select “cartilage” array				
BMP-4	CD109	EphA2	Ephrin-A4	FLRG	Aggrecan	bFGF	bIG-H3	BMP-2	BMP-5
Flt-3L	FSH	IFNg	IL-12 p40	IL-17	BMP-7	BMP-8	BMP-9	BMPR-IA	BMPR-IB
IL-20	IL-23	IL-23 R	ILT2	MCSF	BMPR-II	CHI3L1	FGF-4	FGF-6	FGF-9
M-CSF RA	MIP-1a	PTH	RANK	TGF-b1	IL-17F	Leptin	LRP-6	Lumican	Matrilin-3
TLR3	TLR4	TNFa	TRANCE	TREM-2	MBL	MMP-13A	NOV	sFRP-3	Thrombospondin-5

a The maps are broken down into the “Human Inflammation Array GS3”, “Human Immune Response Array GS1”, “Custom G-series Select ‘Osteoclast’ Array”, and the “Custom G-series Select ‘Cartilage’ Array”.

### Data Pre-Processing

Once processed, each array was loaded into Genepix 4000B Microarray scanner (Molecular Devices) and imaged using the “GenePix® Pro 6 Microarray Acquisition & Analysis Software”. Proteins for each array type (Table 2) were analyzed in quadruplicate. The florescent signal intensity values from ‘F532 Mean – B532′ for a given protein were included if the value of the spot did not exceed 1.2x the median value of the collective spots for a protein.^37^ The average number of replicates included for the Inflammation, immune response, cartilage, and osteoclast arrays across the entire data set were 3.5, 3.4, 3.6, 3.5 respectively (max is *n* = 4). All spots which met the inclusion criteria were averaged and a log2 transform was applied. Final values were normalized (eq 1) to the average of two control samples (Human Serum Cellect^TM^, Fisher BioReagents, BP2657–100) run on opposite ends of each array. All data processing took place in Spyder IDE using Python 3.12 and Microsoft Excel.

1 $$\mathrm{normalized}\;\mathrm{samples}=\frac{\mathrm{averaged}\;\mathrm{raw}\;\mathrm{sample}}{\mathrm{averaged}\;\mathrm{raw}\;\mathrm{control}}$$

### Statistical Analysis

Samples were split into cohorts based on the origin of the BMAC samples (iliac crest or humeral head), patient sex (male or female), and patient smoking status (current, former, or never-smoker). To assess normality the Shapiro-Wilk test was used. Two-group sample comparisons were performed using the nonparametric Mann–Whitney U test and three-group sample comparisons used a Kruskal–Wallis test with Dunn’s post hoc analysis. All samples were subject to false discovery corrections using the Benjamini-Hochberg method to control multiple statistical testing. For all tests a significance threshold of *p* < 0.05 was used.

### Results

A statistical analysis was conducted on the BMAC data set comprised of 120 patient observations screening 109 proteins per patient.

The BMAC data set was initially stratified by male (*n* = 55) and female (*n* = 65) patients. The normality of each protein in the data set (*n* = 109) was assessed using a Shapiro-Wilk test where a *p*-value < 0.05 would suggest a given protein does not have a normal distribution. Given that more than half of the proteins in the data set test as having a non-normal distribution a Mann–Whitney U (MWU) test was used as a nonparametric two-group comparison test. Benjamini-Hochberg false discovery rate (FDR) corrections were applied to the raw p-values obtained from the MWU testing to control falsely identified significant proteins (Type 1 error). Of the 109 proteins examined, only three proteins being BMP-5, BMPR-IA, and Matrilin-3 had a raw p-value less than 0.05, however when FDR corrections were applied those proteins were no longer statistically. This process was repeated with the male–female groups, except, each group was further stratified by their BMAC origin site (humeral head or iliac crest). The results can be seen in Table 3.

**Table 3:** Statistical Analysis for BMAC Data Set Patients Stratified by Male (M) and Female (F) with Respect to Sample Extraction Site (Humeral Head, Iliac Crest, Both)^a^

data set	group Size (M/F)	shapiro non-normal (M/F)	MWU raw sig proteins (*p*< 0.05)	MWU FDR sig proteins (*p*< 0.05)	significant proteins (Raw*p* < 0.05)
iliac crest (77)	31 M/46 F	56/76 → 49 overlap	11	0	BMP-5, BMPR-IA, FGF-4, FGF-6, matrilin-3, Thrombospondin-5, bIG-H3, CRP, CD109, Ephrin-A4, IL-20
humeral head (43)	24 M/19 F	51/63 → 33 overlap	0	0	—
both (120)	55 M/65 F	77/89 → 70 overlap	3	0	BMP-5, BMPR-IA, Matrilin-3

a Shapiro-Wilk test, Mann–Whitney U (MWU) test, and Benjamini-Hochberg false discovery rate corrections for MWU results as well as significant proteins (raw *P* < 0.05) are listed in columns 3,4,5, and 6 respectively.

The BMAC data set was stratified by patient smoking status being, smoker (*n* = 24), former smoker (*n* = 31), or never smoker (*n* = 65). Again, the Shapiro-Wilk test was used to assess the normality of each group (*p* < 0.05); for 109 total proteins, the smoker group had 51, former smokers had 65, and never smokers had 85 proteins with a non-normal distribution. The Kruskal–Wallis (KW) test was used as a nonparametric multigroup (2 < *n*) comparison test to identify any significant differences between the smoker, former smoker, and the never smoker groups. The KW test identified BMPR-II, G-CSF, IL-12p70, IL-17, and TRANCE as statistically significantly different within the test groups. A Dunn’s posthoc test was applied as a pairwise comparison to identify the location of the significant proteins identified by the KW test. False discovery rate (FDR) correction using the Benjamini–Hochberg method was applied to both the Kruskal–Wallis *p*-values (across all proteins) and the Dunn post hoc *p*-values (within each protein’s pairwise comparisons) to control for multiple hypothesis testing. This procedure was performed twice more on the data set, however, the BMAC extraction site (humeral head or iliac crest) was taken into account. The results are summarized in Table 4.

**Table 4:**
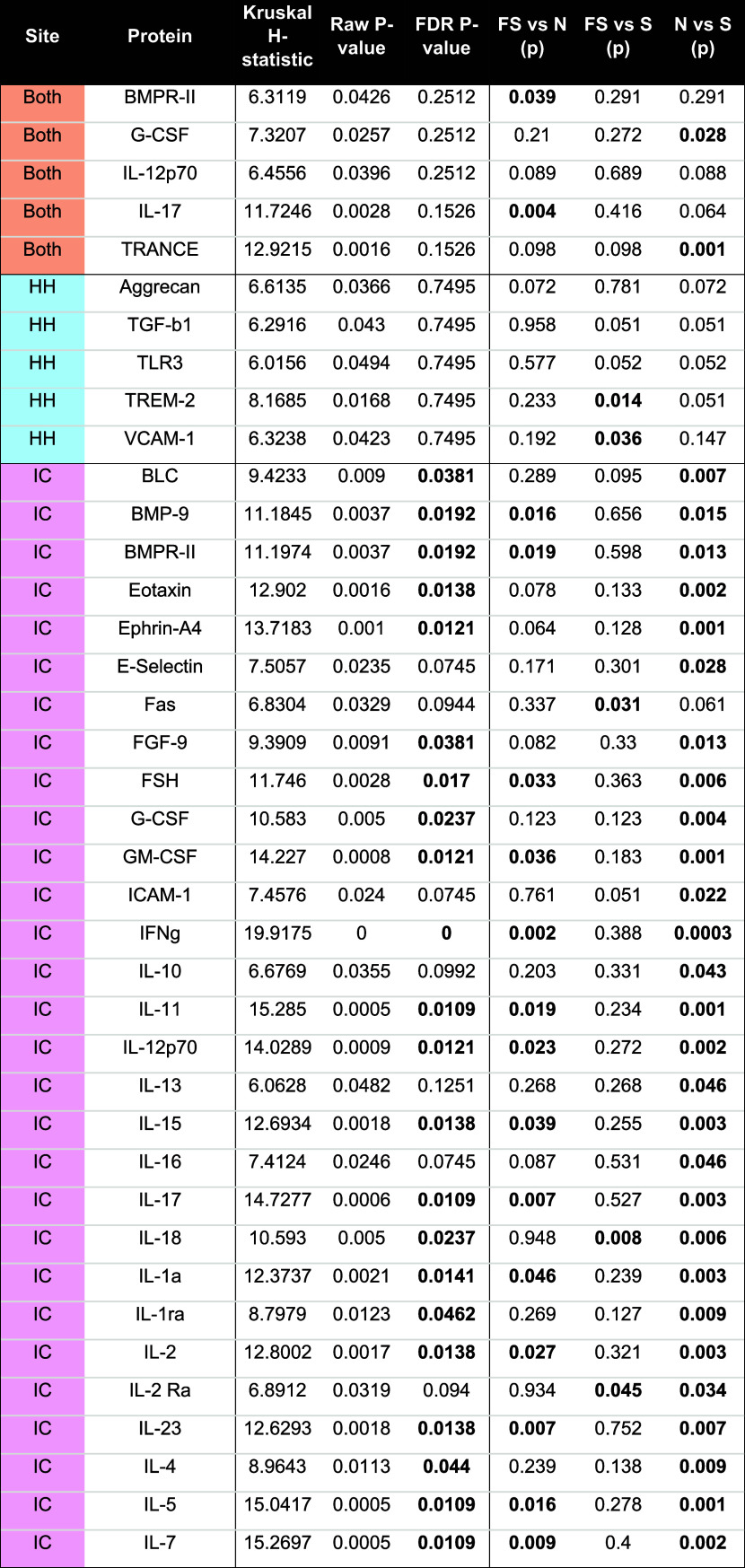

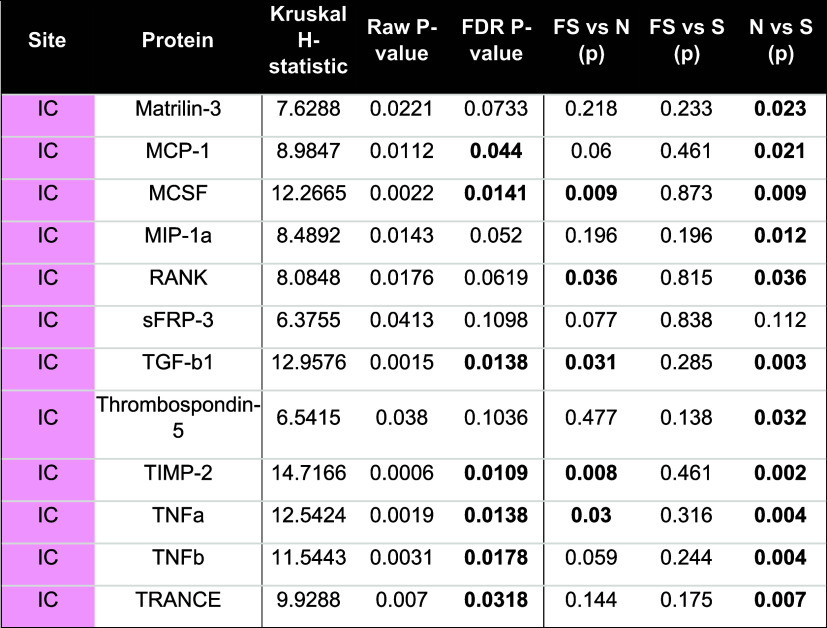
Statistical Analysis for BMAC Data Set Patients Stratified by Smoking Status with Respect to Sample Extraction Site (Humeral Head, Iliac Crest, Both)^a^,^a^

a Summarized results for all Kruskal–Wallis and Dunn’s post hoc statistical analysis. All proteins reported had a ‘Raw P-value’ of *p* < 0.05. A false discovery rate (FDR) correction using the Benjamini-Hochberg method; All proteins whose ‘FDR P-value’ was *p* < 0.05, were statistically significant. The following three columns: FS vs N’, FS vs S’, and ‘N vs S’ were the result of Dunn’s post hoc test, values listed in the columns are p-values where *p* < 0.05 was statistically significant.

b Note that FS = Former Smokers, S = Smokers, *N* = Never smokers.

Following the statistical analysis, proteins were analyzed by comparing their normalized RFU’s with respect to either age, BMI, signal sample density, based on sex or smoking cohorts. Proteins analyzed based on sex specific differences were done with respect to whether or not the proteins raw P-value was *p* < 0.05 (Table 3). Proteins analyzed based on smoking were selected with respect to the FDR corrected P-value being *p* < 0.05 (Table 4).The full list of proteins found to be statistically significant are in the supplemental image file (Figures S1–S3 for Sex and Figures S4–S11 for Smoking).

## Discussion

The BMAC data set is currently composed of 120 patient observations, each described by 109 unique proteins; these proteins were analyzed using one of four antibody microarrays (Table 2). The choice for using the inflammation, immune response, osteoclast, and chondrogenic arrays comes from their analyte diversity and presence of key proteins that other works may describe as integral to the nature of BMAC’s regenerative properties. Notable examples include, BMP-2, BMP-7, IL-1ra, TGF-B1, VEGF, and IL-8.^19,38^ Although other arrays may have been informative in isolation, our selection captures many of the canonical pathways associated with BMAC. Notably, the design of this study allows for future horizontal expansion (additional proteins) and vertical expansion (additional patient observations). Stated previously, the role of patient demographic factors is largely understudied in the context of BMAC. Our goal was to identify if and how a patients age, sex, BMI, or smoking status may play a role in the proteomic composition of BMAC.

Our analyses looked at the BMAC samples in one of two ways, stratified by bone marrow source or grouped as a whole. The rationale for bone marrow source stratification was derived from our previous work which identified several key differences between the BMAC proteomes depending on the location of the bone marrows extraction.^39^ Further stratifications were used to analyze patients either by biological sex or smoking status.

A Shapiro-Wilk’s test was used to assess the normality of each protein being analyzed with respect to each division of groups. As expected, many of the proteins failed the Shapiro-Wilk test, obtaining a p-value of *p* < 0.05, meaning the protein in question lacked a normal distribution. This information is important in the way of determining what types of statistical tests are appropriate for the data set. Given the large mixture of normally and non-normally distributed proteins, we opted for a nonparametric series of statistical tests (Tables 3 and 4).^40,41^

We first examined patients by their biological sex. The normality of each group was mixed and therefore a Mann–Whitney U (MWU) test was used to perform a two-group comparison. The unadjusted or raw MWU results showed that 11 proteins were identified as statistically significantly different between males and females whose BMAC sample originated from the iliac crest, 0 proteins for the humeral head samples, and 3 proteins with all samples included (humeral head + iliac crest). In total 11 unique proteins were characterized as statistically significant these proteins being: BMP-5, BMPR-IA, FGF-4, FGF-6, Matrilin-3, Thrombospondin-5, bIG-H3, CRP, CD109, Ephrin-A4, and IL-20.

Notably, none of these proteins survived the Benjamini-Hochberg FDR corrections, all proteins having an adjusted p-value of *p* > 0.05. Contextually, FDR corrections are often used in -omics analyses as a means of controlling for false significant features (proteins, genes, biomarkers, etc). However, given the smaller sample size of our data set it is important to take protein functions into account when determining the magnitude of their effect on a system.

Our results suggest that biological sex differences do manifest in the BMAC proteome albeit in a subset of proteins whose functions directly relate to bone and cartilage development in the way of BMPs, FGFs, and extracellular matrix proteins like Matrilin-3, Thrombospondin-5, and bIG-H3.^42–46^

To analyze the smoking cohorts, we stratified patients by bone marrow source (iliac crest, humeral head, or both) in addition to their smoking statuses (current, former, or never smokers). The normality of each protein (Table 2) within the cohorts was assessed using the Shapiro-Wilk test. Following this, a Kruskal–Wallis′ (KW) test was performed as the nonparametric equivalent to an ANOVA; this test enabled us to identify that there are differences between *n* > 2 groups of samples and produces an H-statistic which relates to how big those differences are. The KW test identified 51 proteins across 3 testing groups which had a raw p-value of *p* < 0.05. When Benjamini-Hochberg FDR corrections were applied to these results, only 29 of those proteins survived; all proteins which contained a significant FDR adjusted p-value where *p* < 0.05 belonged to the iliac crest smoking cohort (Table 4). Within these 29 proteins, we were able to use Dunn’s post hoc testing (with FDR corrections) to identify where the differences were between smokers, former smokers, and never smokers.

Smoking status was associated with substantial alterations in the BMAC proteome, with 29 proteins in the iliac crest cohort showing FDR-corrected significant *p*-values. Of the 29 proteins, 18 were differentially expressed between smokers and never smokers, with former smokers often exhibiting moderate profiles. Notably smoking patients from the iliac crest cohort demonstrated a pro-inflammatory skew, having elevated levels of catabolic mediators (e.g., IL-17, TNF-a, IL-1a) and reduced anti-inflammatory proteins (e.g., IL-1ra, IL-4). Osteo- and chondrogenic factors such as BMP-9, FGF-9, TGF-β1 showed lower levels in the smoking patients, suggesting a potentially impaired regenerative profile (Tables 4 and 5).

**Table 5:** Tabulated FDR Significant Proteins with General Function and Classifications^a^,^b^

protein	function	classification	reference
*IL-1ra	antagonist of IL-1, anti-inflammatory	anti-inflammatory	^12,23^
*IL-4	anti-inflammatory promotes Th2 responses	anti-inflammatory, immunomodulatory	^19^
TIMP-2	inhibits MMPs, regulates extracellular matrix turnover	chondrogenic	^38^
*FGF-9	promotes chondrocyte proliferation	chondrogenic	^38,43^
TGF-β1	anti-inflammatory, promotes chondrogenesis and fibrosis	chondrogenic, anti-inflammatory	^12,19,22,42^
IL-5	stimulates eosinophils, associated with allergic responses	immunomodulatory	^19^
IL-7	lymphocyte development and survival	immunomodulatory	^19^
IL-2	T-cell growth factor, promotes immune activation	immunomodulatory	^19^
*G-CSF	stimulates neutrophil production, immunomodulatory	immunomodulatory	^19^
*BLC (CXCL13)	B-cell chemokine, involved in lymphoid organization	immunomodulatory	^19^
*Ephrin-A4	cell signaling, angiogenesis, bone remodeling	osteogenic	^19,20^
FSH	hormone with potential bone resorption effects	osteogenic	^19^
BMP-9	promotes osteoblast differentiation	osteogenic	^42^
BMPR-II	receptor for BMPs, involved in osteogenesis	osteogenic	^42^
*TRANCE (RANKL)	essential for osteoclast differentiation	osteogenic	^19,20^
IL-11	promotes osteoblast differentiation, anti-inflammatory in some contexts	osteogenic, immunomodulatory	^19^
M-CSF	stimulates osteoclast formation, regulates macrophage function	osteogenic, immunomodulatory	^19^
IL-17	pro-inflammatory cytokine, promotes bone resorption, Th17 responses	pro-inflammatory	^19^
IL-12p70	promotes Th1 responses, pro-inflammatory	pro-inflammatory	^19^
*Eotaxin	chemokine for eosinophils, involved in allergic inflammation	pro-inflammatory	^19^
IL-23	promotes Th17 responses, pro-inflammatory	pro-inflammatory	^19^
TNF-α	pro-inflammatory, promotes bone resorption	pro-inflammatory	^19,23^
IL-1α	pro-inflammatory, promotes cartilage degradation	pro-inflammatory	^19,23^
*TNF-β	pro-inflammatory, similar to TNF-α	pro-inflammatory	^19^
**IL-18	pro-inflammatory, promotes IFN-γ production	pro-inflammatory	^19^
*MCP-1	recruits monocytes, pro-inflammatory	pro-inflammatory	^19,21^
IFN-γ (IFNg)	pro-inflammatory cytokine, promotes Th1 responses, inhibits osteogenesis	pro-inflammatory, immunomodulatory	^19,21^
GM-CSF	stimulates myeloid cell proliferation, pro-inflammatory	pro-inflammatory, immunomodulatory	^19,21^
IL-15	stimulates NK and T cells, pro-inflammatory	pro-inflammatory, immunomodulatory	^19^

a Table 5. Tabulation of all proteins whose FDR-adjusted p-value (listed in Table 4) was *p* < 0.05. All proteins listed contain their name, general function, and classification.

b Note that all proteins in this table are from the Iliac Crest Kruskal–Wallis testing group. Items preceded by * = Different between Smokers and Never Smokers only, ** = Different between Smokers and Never Smokers as well as Former Smokers and Smokers. Items with no prefix were different between both the Smokers and Never Smokers as well as Former Smokers and Never Smokers.

It is important to note the interplay between dysregulated levels of proteins in the bone marrow. For example, IL-1ra being down-regulated in smoking patients while IL-1a is simultaneously up-regulated may contribute to the catabolic environment. Should a protein like TRANCE (RANKL) also be upregulated, this could promote greater levels of osteoclast activity, which may mirror smoking-associated bone loss. Likewise, reduced expression of proteins like TGF-β1 and BMP-9 would align with studies which link cigarette smoke exposure (CSE) to impair osteogenesis.^34^ CSE has also been shown in mice to reduce chondrocyte viability, proliferation, and matrix formation in both a dose and time dependent manner.^47^ Additionally, a 2025 retrospective clinical study identified that smoking was directly associated with delayed bone union and increased infection rates during lower extremity reconstructions, clinical manifestations that closely coincide with the pro-inflammatory and antianabolic fluctuations of protein expression levels observed on our arrays.^48^

Of particular interest is the site-specific effects which smoking appears to have; Based on our statistical analysis, the iliac crest cohort appears to have a greater degree of variance between patent Smoking statuses’. We found the lack of differentiation between smoking status in the humeral head cohort surprising for a few reasons; Namely, there is a link between a patient smoking and rotator cuff injury.^49–52^ Through a combination of fatty infiltration^53,54^ of the rotator cuff based combined with the plethora of fat-soluble compounds in cigarette smoke,^55^ we had expected a larger observable difference between patients in the humeral head cohort than actually observed.

Based on our previous work with this data set, we established that the iliac crest and humeral head proteomes are different enough to be mathematically distinguishable from one another.^39^ However, when we apply a deeper level of stratification and subsequently shrink the sample sizes for the humeral headgroup in particular, we appear to lose the ability to make distinctions between said stratifications. We believe both from a biological and statistical standpoint that a greater number of humeral head samples would give us a much greater insight into whether the lack of statistically significant differences between smoking statuses are truly not present. Despite the results of the statistical tests, we do have domain knowledge regarding the effects of smoking that would suggest that we should see some observable differences in the humeral head samples.

When interpreting the results presented in this work it is crucial to understand the limitations of the results within the context of the applied methodology. The statistical power associated with subgroup stratification. Initially, the BMAC data set was composed of 120 observations, however, after dividing those by bone marrow source, smoking status, and biological sex the actual volume of samples tested per group was much smaller. These smaller subgroup sizes likely limit the sensitivity for detecting subtle proteomic differences and may have contributed to instability in some statistical comparisons following the false discovery rate correction. This effect is reflected by several proteins exhibiting raw statistical significance yet did not survive the FDR adjustments. Accordingly, negative findings, particularly in the humeral head cohort should be evaluated from both a biological standpoint in conjunction with the statistical information presented. Larger future cohorts will be necessary to improve the statistical power and stabilize the group comparisons through the stratification process.

Furthermore, this study relied solely on the use of antibody microarrays. The antibody microarray enables targeted high-throughput screening against numerous analytes in parallel, providing a relative fluorescence-based quantification rather than a true quantitative concentration measure as seen in something like an ELISA complex. We aimed to maximize the semiquantitative nature of the arrays by being consistent with the total amount (g) of protein loaded into each array per patient. Despite this, measured signal intensities may be influenced by antibody specificity, epitope affinity, saturation effects, and potential cross-reactivity between structurally related proteins. These factors may limit dynamic ranges and sensitivity for low-abundance analytes. Consequently, the observed proteomic differences should be interpreted as relative expression trends rather than definitive concentration changes. Additionally, one of the largest limitations of this work is not having access to more details around the former smokers (in all cohorts), we do not have access to the duration of time patients stopped smoking, nor the volume at which they were smoking; Future studies should seek to stratify their patients using those metrics when possible because there is a very strong possibility that the differential protein expression between former-smokers and the other groups may have been far more pronounced. Additionally, while the modulation of proteins in isolation are apparent with respect to the bone marrow site and smoking status, the interactions between multiple modulated proteins would require in vitro validation for a true causal pathway.

## Conclusion

This study demonstrates that patient demographic factors, namely smoking, coincided with altered proteomic profiles of BMAC. We observed the largest differences in the iliac crest cohort of patients, but BMAC samples originating from the iliac crest and humeral head were profiled. In the iliac crest cohort, anabolic growth factors including TGF-β1 and BMP-9 were downregulated and pro-inflammatory cytokines (IL-17, TNF-a) were upregulated; These findings ultimately suggest that smoking may adversely influence the regenerative proteomic profile of BMAC. Former smokers exhibited moderate smoking proteomic profiles when compared to their smoking counterparts and we observed the proteomic profile of those former smokers to be more in line with the nonsmoking groups.

To properly evaluate the effect of smoking cessation on the BMAC proteomic profile, one would require the length of time a patient has stopped smoking and volume of cigarettes consumed prior to cessation (e.g., packs consumed per unit time). Without those key insights, any evaluations on the effects of smoking cessation would be speculative at best. The results we present fall into the latter.

Additional future work should focus on expanding the humeral head cohort, as increased sample sizes may improve statistical power and help clarify whether smoking-associated proteomic alterations are present but underpowered in this anatomical subgroup. Further mechanistic and in vitro validation studies will also be necessary to better define how coordinated changes in inflammatory, osteogenic, and chondrogenic proteins influence the biological activity of BMAC.

Despite these limitations, the findings presented in this work provide evidence that patient demographic factors, particularly smoking status, are associated with observable alterations in the BMAC proteome and may contribute to variability in the biological characteristics of orthobiologic preparations. Overall, these findings support the importance of patient stratification by smoking status in clinical BMAC applications and highlight the potential value of smoking cessation prior to treatment (Figure 1).

**Figure 1:**
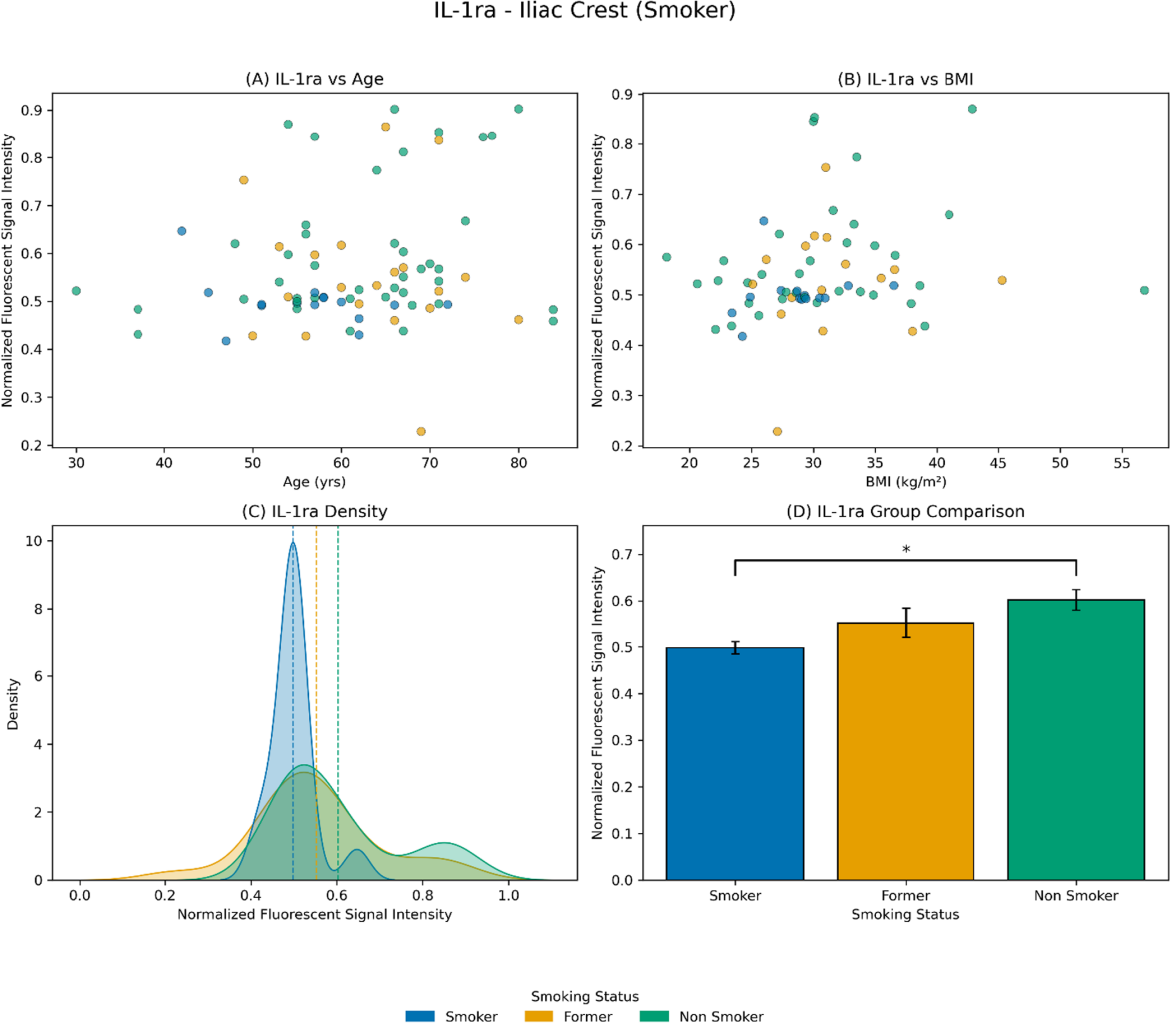
Smoking analysis of IL-1ra. Multipanel visualization of IL-1ra expression in iliac crest BMAC samples stratified by smoking status. Individual data points are colored by smoking group: nonsmoker (N), smoker (S), and former smoker (FS). (A) Scatter plot showing the relationship between normalized fluorescent signal intensity and age. (B) Scatter plot showing the relationship between normalized fluorescent signal intensity and BMI. (C) Kernel density distributions of IL-1ra expression across smoking groups, with dashed vertical lines representing group means. Density plots illustrate the relative distribution and variability of signal intensity within each cohort. (D) Group comparison of mean normalized fluorescent signal intensity ± SEM across smoking categories. Statistically significant differences between a group are denoted by the asterisk (*).

## Supplementary Material



## Data Availability

The data underlying this study is openly available in Mendeley Data at https://data.mendeley.com/datasets/9sykj5s75c/1, DOI:10.17632/9sykj5s75c.1

## Notes

Extension of Figure 1 which contains a combined analysis of how patients age and BMI related to a given protein′s expression levels; the distribution of those expression levels, and a bar chart looking at those expression levels with respect to smoking (or sex); these are summarized below and further details are provided following; supporting Figures 1,2, and 3 evaluate proteins based on sex differences with respect to the statistically significant proteins with a **raw** *p* **< 0.05**, a list of these proteins can be seen in Table 3 in the ‘Significant proteins’ column; supporting Figures 4–11 evaluate proteins based smoking differences with respect to the statistically significant **FDR** *p* **< 0.05**; a list of these proteins can be seen in Table 4 under the “FDR P-Value” column as the **bolded values**; Table 5 also contains the same proteins. The table of contents provides further details: **Table of Contents**; sex specific analysis of: BMP-5, BMPR-IA, FGF-4, FGF-6 (Figure S1); sex specific analysis of: CD109, Ephrin-A4, IL-20 (Figure S2); sex specific analysis of: Matrilin-3, Thrombospondin-5, bIG-H3, CRP; smoking specific analysis of: BLC, BMP-9, BMPR-II, Eotaxin (Figure S4); smoking specific analysis of: Ephrin-A4, FGF-9, FSH, G-CSF (Figure S5); smoking specific analysis of: GM-CSF, IFNg, IL-11, IL-12p70 (Figure S6); smoking specific analysis of: IL-15, IL-17, IL-18, IL-1a (Figure S7); smoking specific analysis of: IL-1ra, IL-2, IL-23, IL-4 (Figure S8); smoking specific analysis of: IL-5, IL-7, MCP-1, MCSF (Figure S9); smoking specific analysis of: TGF-b1, TIMP-2, TNFa, TNFb (Figure S10); smoking specific analysis of: TRANCE (Figure S11) (PDF)
