# Supplementary material for: Demographic Factors Affect the BMAC Proteome
Source: J Proteome Res. 2026 Jul 27;25(8):3835–44. doi: 10.1021/acs.jproteome.5c00804 (PMC13459544; doi:10.1021/acs.jproteome.5c00804)
Supplement: Supplementary file 1 [file pr5c00804_si_001.pdf]

Title: Demographic Factors Affect the BMAC Proteome

Colin Herna[1], Madison Cipriani[1], Ranjan Sachdev[2], Sabrina Jedlicka[1]*

1. Lehigh University, Bioengineering 5 E. Packer Ave, Bethlehem, PA, US 18015
2. Sachdev Orthopaedics, 3735 Nazareth Rd, 302 A, Easton, PA 18045

Corresponding Author Contact:

Sabrina Jedlicka PhD

Email: SSJ207@lehigh.edu

Phone: 610-758-4425

Address: Lehigh University, 5 E. Packer Ave, Bethlehem, PA, US 18015

Table of Contents:

Figure S1. Sex specific analysis of: BMP-5, BMPR-IA, FGF-4, FGF-6

Figure S2. Sex specific analysis of: CD109, Ephrin-A4, IL-20

Figure S3. Sex specific analysis of: Matrilin-3, Thrombospondin-5, bIG-H3, CRP

Figure S4. Smoking specific analysis of: BLC, BMP-9, BMPR-II, Eotaxin.

Figure S5. Smoking specific analysis of: Ephrin-A4, FGF-9, FSH, G-CSF.

Figure S6. Smoking specific analysis of: GM-CSF, IFNg, IL-11, IL-12p70.

Figure S7. Smoking specific analysis of: IL-15, IL-17, IL-18, IL-1a.

Figure S8. Smoking specific analysis of: IL-1ra, IL-2, IL-23, IL-4.

Figure S9. Smoking specific analysis of: IL-5, IL-7, MCP-1, MCSF.

Figure S10. Smoking specific analysis of: TGF-b1, TIMP-2, TNFa, TNFb.

Figure S11. Smoking specific analysis of: TRANCE.

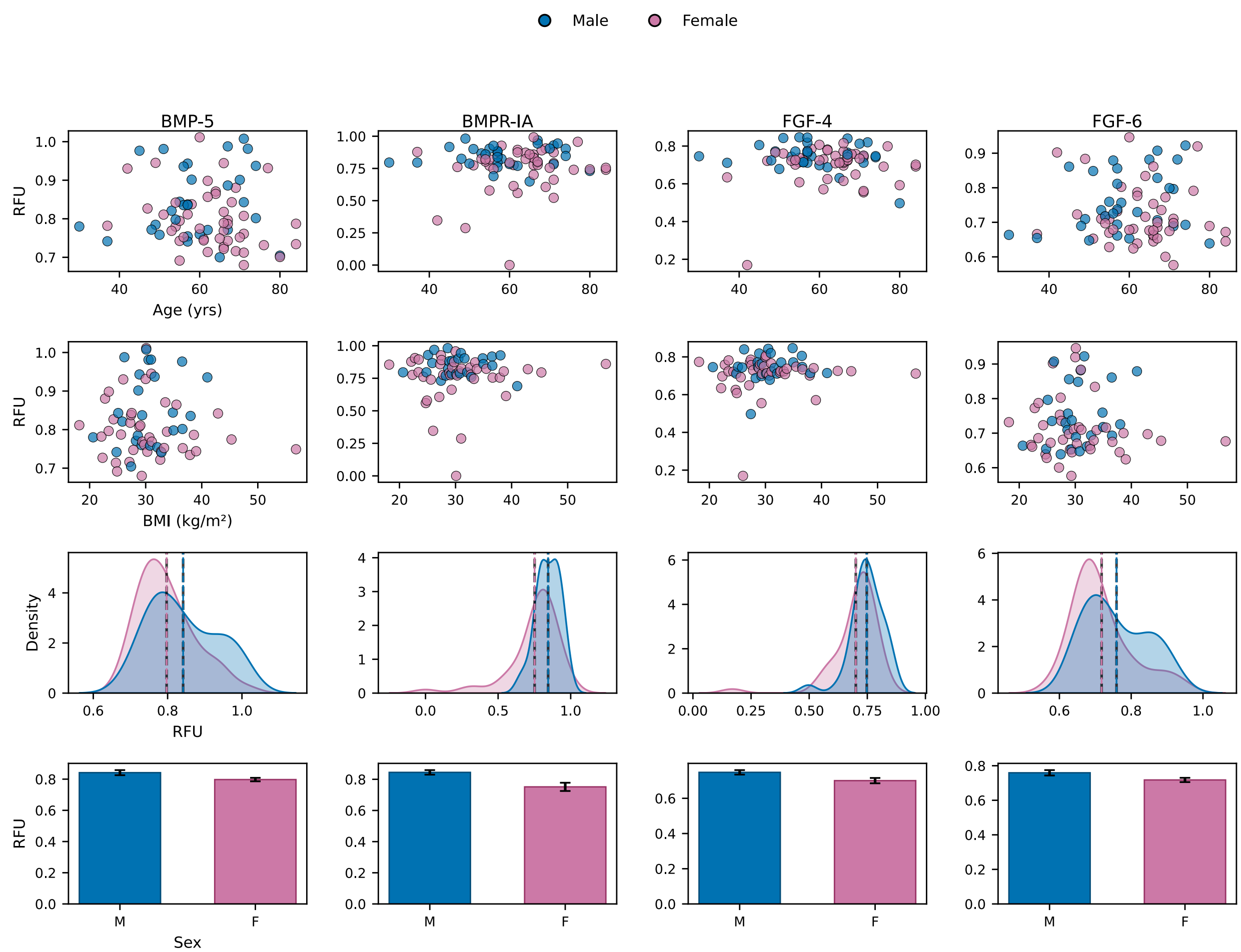

Figure S1. Sex specific analysis of: BMP-5, BMPR-IA, FGF-4, FGF-6. *Statistically significant (raw p<0.05) proteins (4 of 11) identified for males and females: Visualization of the relative normalized fluorescent units (RFU) for each protein. For each protein column (i.e., protein), the subsequent rows depict*

(a) Scatter plot of normalized RFU vs. patient age.
(b) Scatter plot of normalized RFU vs. patient BMI.
(c) Kernel density plot showing distribution of RFU across smoking cohorts, with dashed vertical lines indicating group means, Density plots illustrate the relative distribution and variability of signal intensity within each cohort.
(d) Bar plot of average RFU per smoking status with standard error bars.
Proteins are grouped arbitrarily into sets (e.g., Group 1, Group 2) based on panel layout for figure clarity; these groupings do not represent biological or statistical cohorts.
(D) Group comparison of mean normalized fluorescent signal intensity ± SEM across smoking categories.

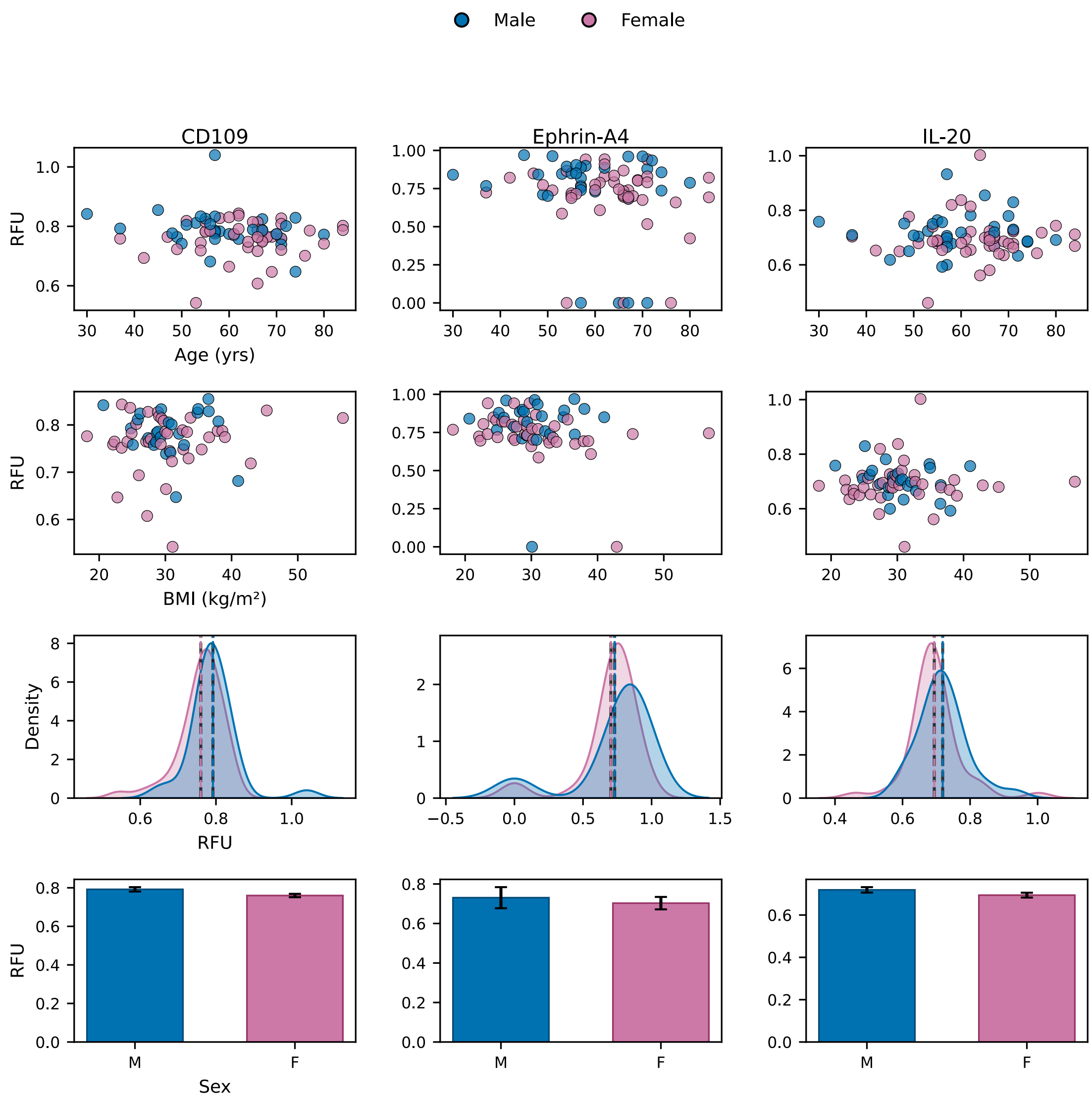

Figure S2. Sex specific analysis of: CD109, Ephrin-A4, IL-20. *Statistically significant (raw p<0.05) proteins (3 of 11) identified for males and females: Visualization of the relative normalized fluorescent units (RFU) for each protein. For each protein column (i.e., protein), the subsequent rows depict*

(a) Scatter plot of normalized RFU vs. patient age.

(b) Scatter plot of normalized RFU vs. patient BMI.

(c) Kernel density plot showing distribution of RFU across smoking cohorts, with dashed vertical lines indicating group means, Density plots illustrate the relative distribution and variability of signal intensity within each cohort.

(d) Bar plot of average RFU per smoking status with standard error bars.

Proteins are grouped arbitrarily into sets (e.g., Group 1, Group 2) based on panel layout for figure clarity; these groupings do not represent biological or statistical cohorts.

(D) Group comparison of mean normalized fluorescent signal intensity ± SEM across smoking categories.

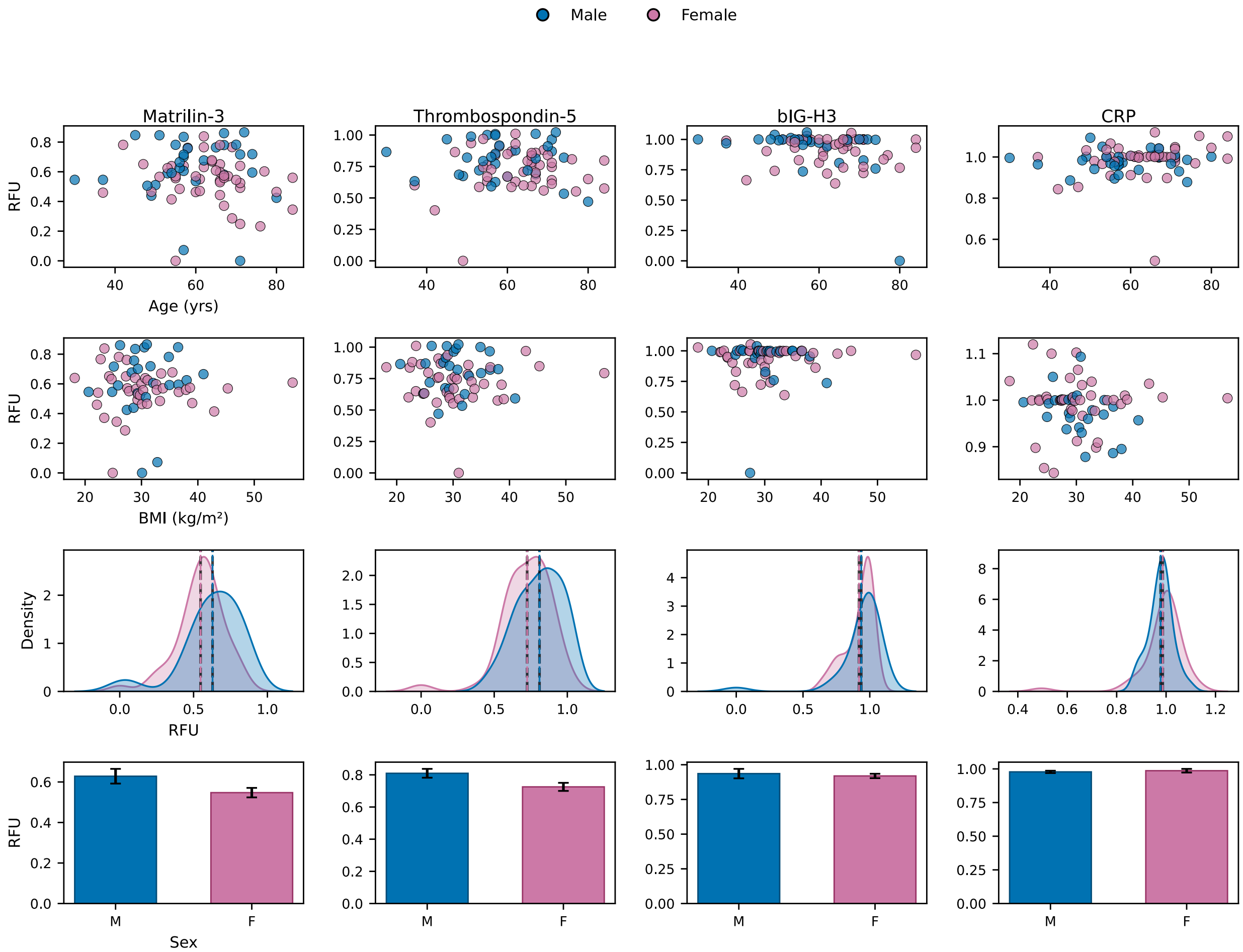

Figure S3. Sex specific analysis of: Matrilin-3, Thrombospondin-5, bIG-H3, CRP.
Statistically significant (raw p<0.05) proteins (4 of 11) identified for males and females: Visualization of the relative normalized fluorescent units (RFU) for each protein. For each protein column (i.e., protein), the subsequent rows depict
(a) Scatter plot of normalized RFU vs. patient age.
(b) Scatter plot of normalized RFU vs. patient BMI.
(c) Kernel density plot showing distribution of RFU across smoking cohorts, with dashed vertical lines indicating group means, Density plots illustrate the relative distribution and variability of signal intensity within each cohort.
(d) Bar plot of average RFU per smoking status with standard error bars.
Proteins are grouped arbitrarily into sets (e.g., Group 1, Group 2) based on panel layout for figure clarity; these groupings do not represent biological or statistical cohorts.
(D) Group comparison of mean normalized fluorescent signal intensity ± SEM across smoking categories.

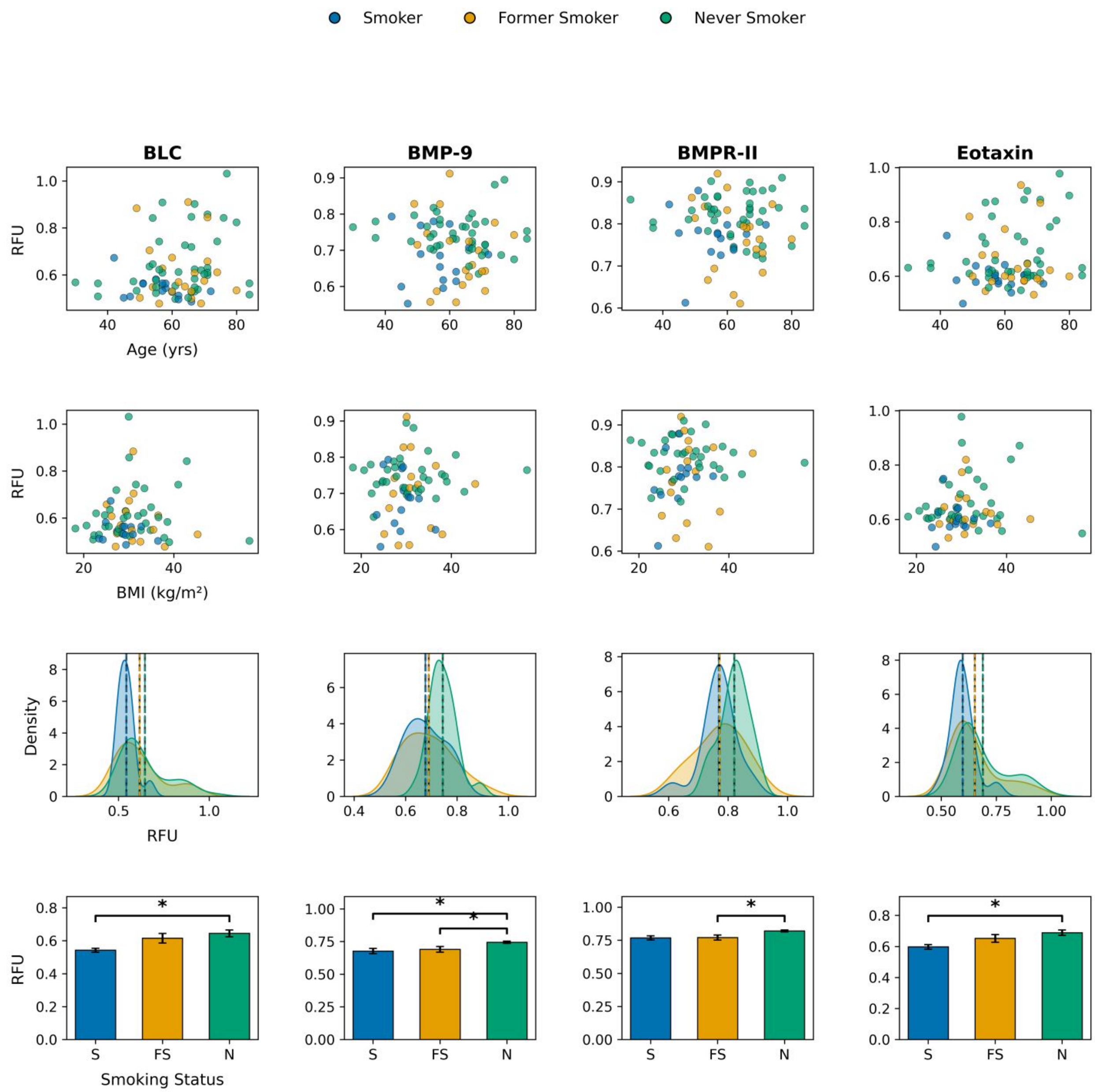

Figure S4. Smoking specific analysis of: BLC, BMP-9, BMPR-II, Eotaxin.
Statistically significant (FDR p<0.05) proteins from Duns Post Hoc analysis: Visualization of the relative normalized fluorescent units (RFU) for each protein (4 of 40). For each protein column (i.e., protein), the subsequent rows depict
(a) Scatter plot of normalized RFU vs. patient age.
(b) Scatter plot of normalized RFU vs. patient BMI.
(c) Kernel density plot showing distribution of RFU across smoking cohorts, with dashed vertical lines indicating group means, Density plots illustrate the relative distribution and variability of signal intensity within each cohort.

(d) *Group comparison of mean normalized fluorescent signal intensity ± SEM across smoking categories. Statistically significant differences between a group are denoted by the asterisk (*).*

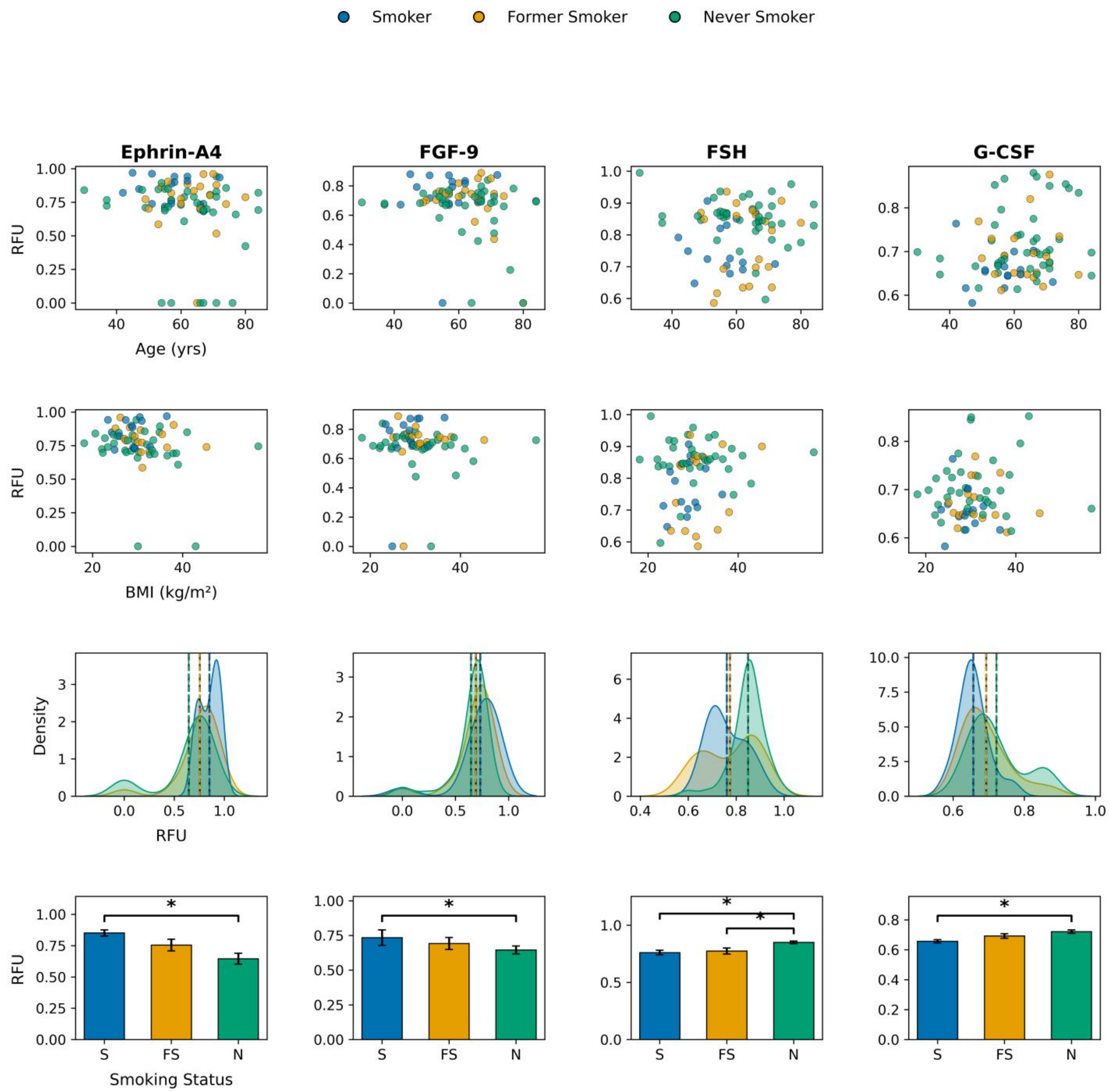

Figure S5. Smoking specific analysis of: Ephrin-A4, FGF-9, FSH, G-CSF.

Statistically significant (FDR p<0.05) proteins from Duns Post Hoc analysis: Visualization of the relative normalized fluorescent units (RFU) for each protein (4 of 40). For each protein column (i.e., protein), the subsequent rows depict

(a) Scatter plot of normalized RFU vs. patient age.

(b) Scatter plot of normalized RFU vs. patient BMI.

(c) Kernel density plot showing distribution of RFU across smoking cohorts, with dashed vertical lines indicating group means, Density plots illustrate the relative distribution and variability of signal intensity within each cohort.
(d) Group comparison of mean normalized fluorescent signal intensity ± SEM across smoking categories. Statistically significant differences between a group are denoted by the asterisk (*).

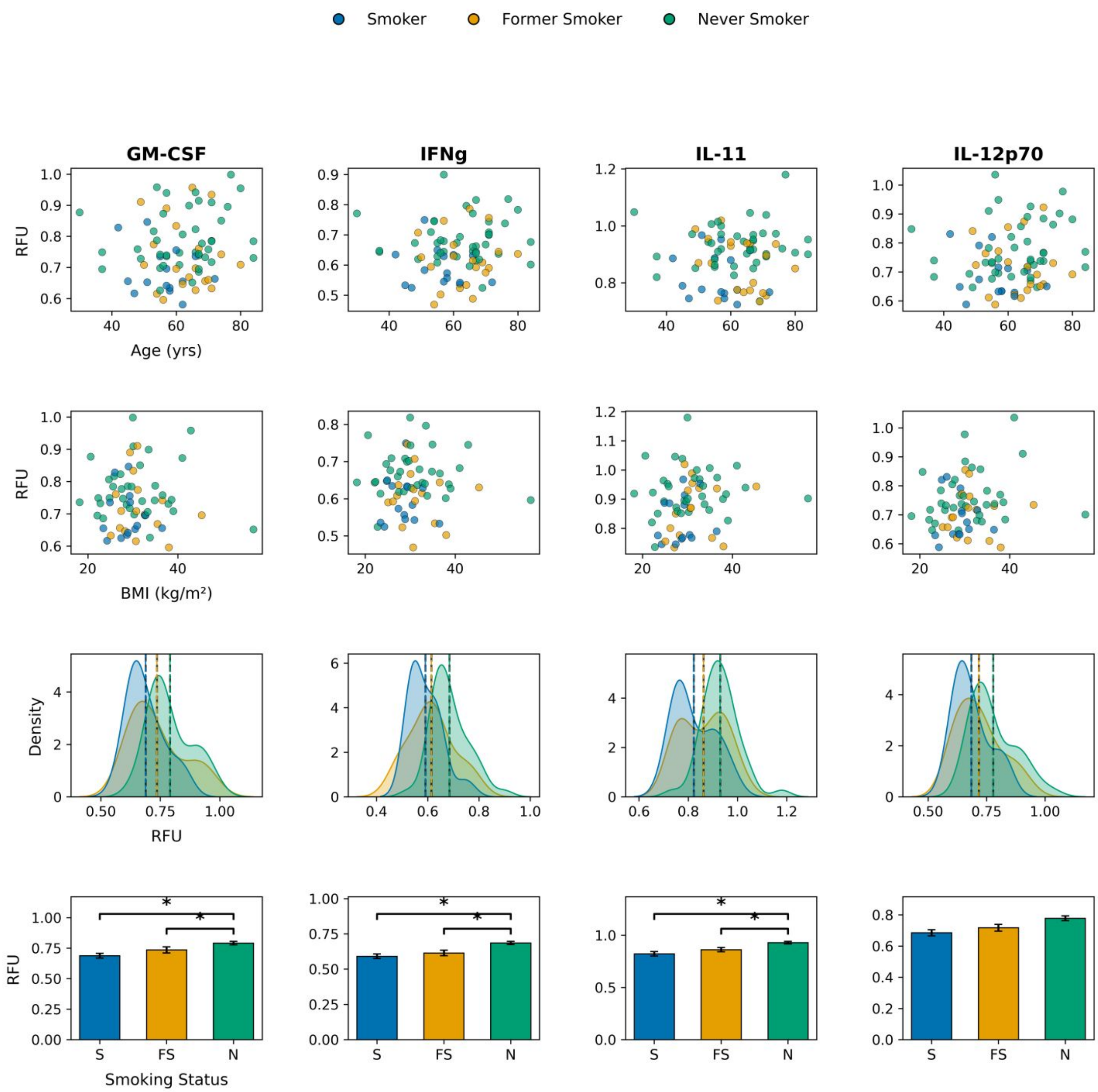

Figure S6. Smoking specific analysis of: GM-CSF, IFNg, IL-11, IL-12p70.
Statistically significant (FDR p<0.05) proteins from Duns Post Hoc analysis: Visualization of the relative normalized fluorescent units (RFU) for each protein (4 of 40). For each protein column (i.e., protein), the subsequent rows depict

(a) Scatter plot of normalized RFU vs. patient age.
(b) Scatter plot of normalized RFU vs. patient BMI.
(c) Kernel density plot showing distribution of RFU across smoking cohorts, with dashed vertical lines indicating group means, Density plots illustrate the relative distribution and variability of signal intensity within each cohort.
(d) Group comparison of mean normalized fluorescent signal intensity ± SEM across smoking categories. Statistically significant differences between a group are denoted by the asterisk (*).

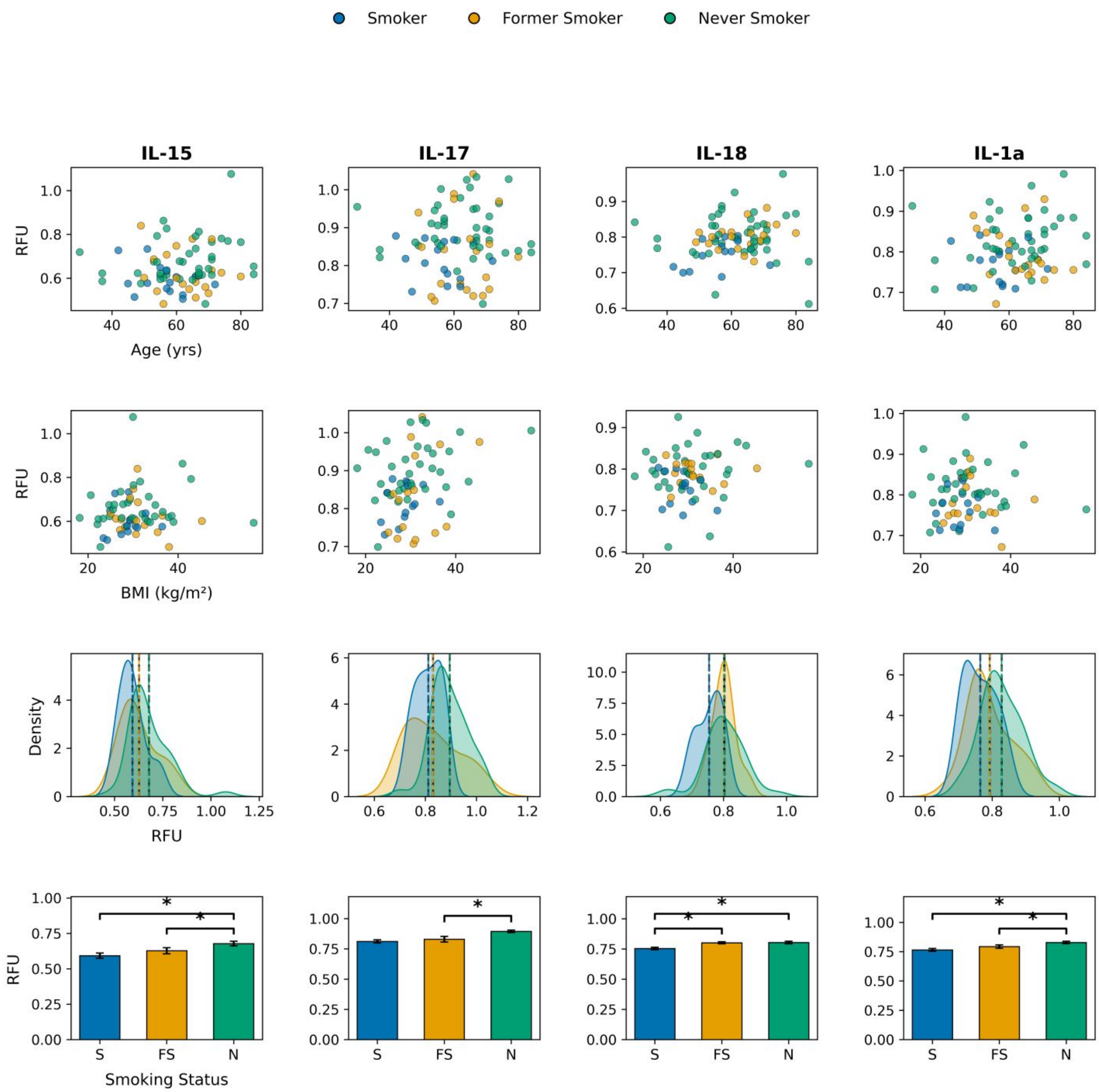

Figure S7. Smoking specific analysis of: IL-15, IL-17, IL-18, IL-1a. *Statistically significant (FDR p<0.05) proteins from Duns Post Hoc analysis: Visualization of the*

relative normalized fluorescent units (RFU) for each protein (4 of 40). For each protein column (i.e., protein), the subsequent rows depict

(a) Scatter plot of normalized RFU vs. patient age.

(b) Scatter plot of normalized RFU vs. patient BMI.

(c) Kernel density plot showing distribution of RFU across smoking cohorts, with dashed vertical lines indicating group means, Density plots illustrate the relative distribution and variability of signal intensity within each cohort.

(d) Group comparison of mean normalized fluorescent signal intensity ± SEM across smoking categories. Statistically significant differences between a group are denoted by the asterisk (*).

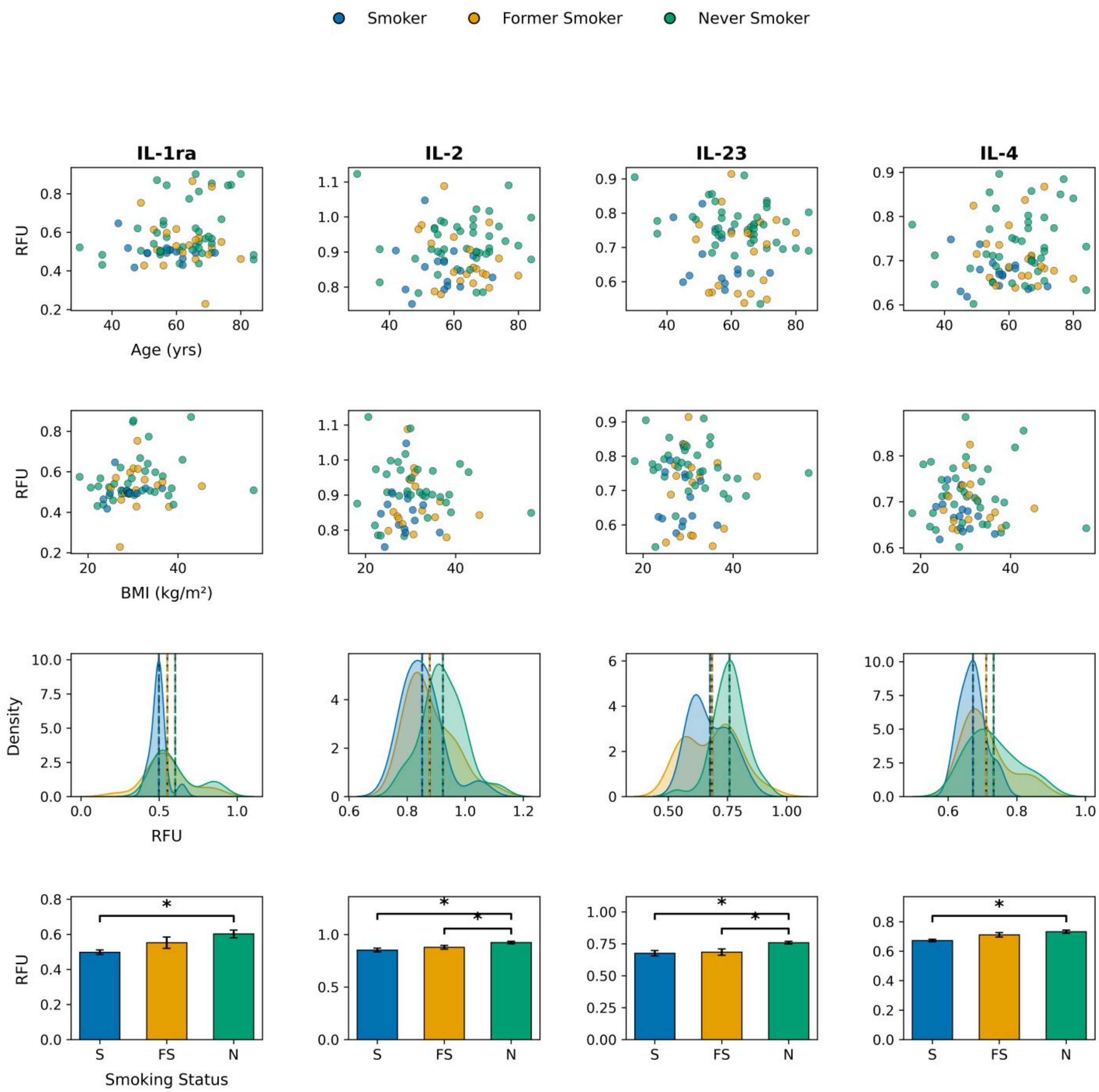

Figure S8. Smoking specific analysis of: IL-1ra, IL-2, IL-23, IL-4. *Statistically significant (FDR p<0.05) proteins from Duns Post Hoc analysis: Visualization of the relative normalized fluorescent units (RFU) for each protein (4 of 40). For each protein column (i.e., protein), the subsequent rows depict*

(a) Scatter plot of normalized RFU vs. patient age.

(b) Scatter plot of normalized RFU vs. patient BMI.

(c) Kernel density plot showing distribution of RFU across smoking cohorts, with dashed vertical lines indicating group means, Density plots illustrate the relative distribution and variability of signal intensity within each cohort.

(d) Group comparison of mean normalized fluorescent signal intensity ± SEM across smoking categories. Statistically significant differences between a group are denoted by the asterisk (*).

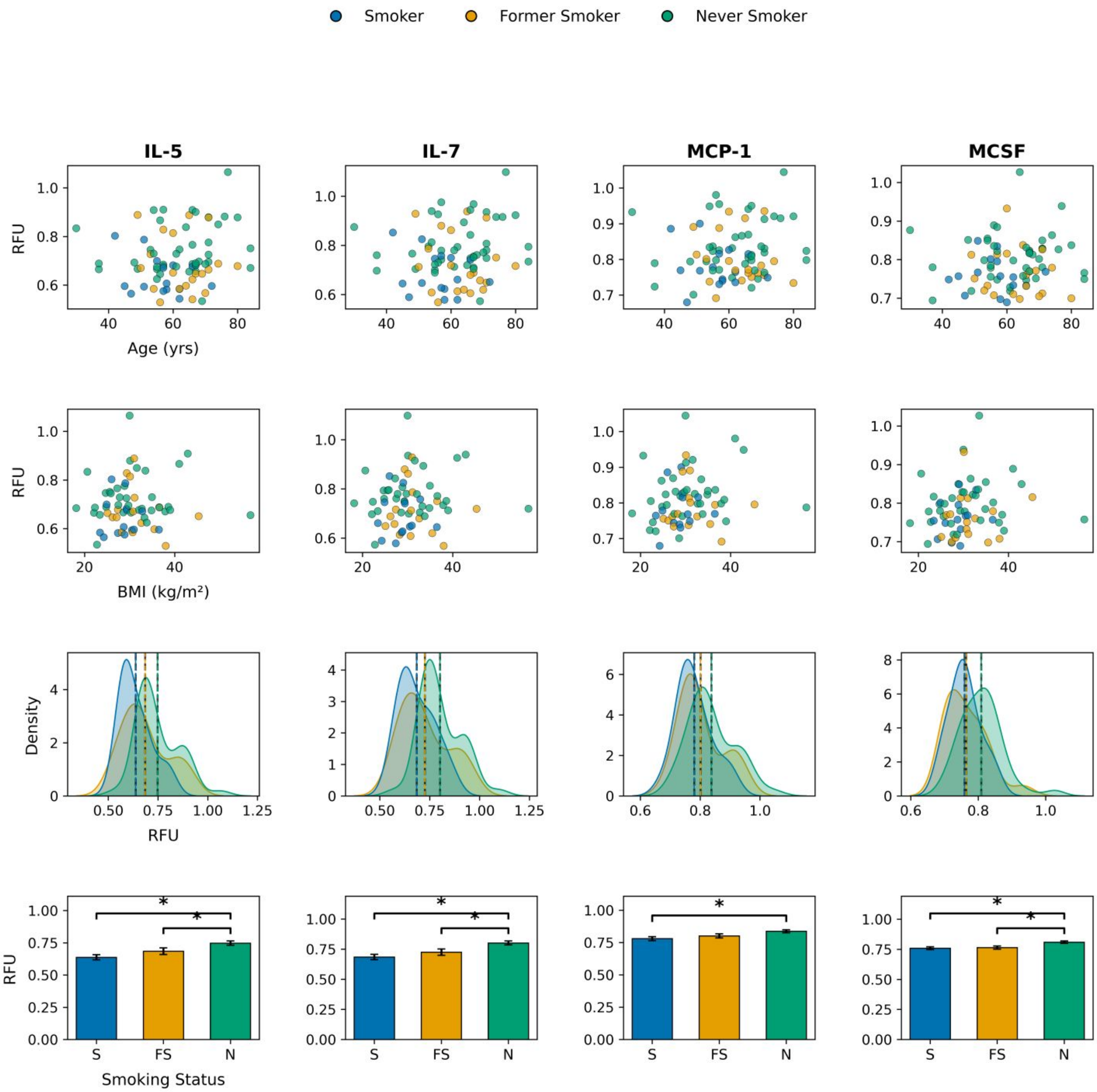

Figure S9. Smoking specific analysis of: IL-5, IL-7, MCP-1, MCSF. *Statistically significant (FDR p<0.05) proteins from Duns Post Hoc analysis: Visualization of the relative normalized fluorescent units (RFU) for each protein (4 of 40). For each protein column (i.e., protein), the subsequent rows depict*
(a) Scatter plot of normalized RFU vs. patient age.
(b) Scatter plot of normalized RFU vs. patient BMI.

(c) Kernel density plot showing distribution of RFU across smoking cohorts, with dashed vertical lines indicating group means, Density plots illustrate the relative distribution and variability of signal intensity within each cohort.

(d) Group comparison of mean normalized fluorescent signal intensity ± SEM across smoking categories. Statistically significant differences between a group are denoted by the asterisk (*).

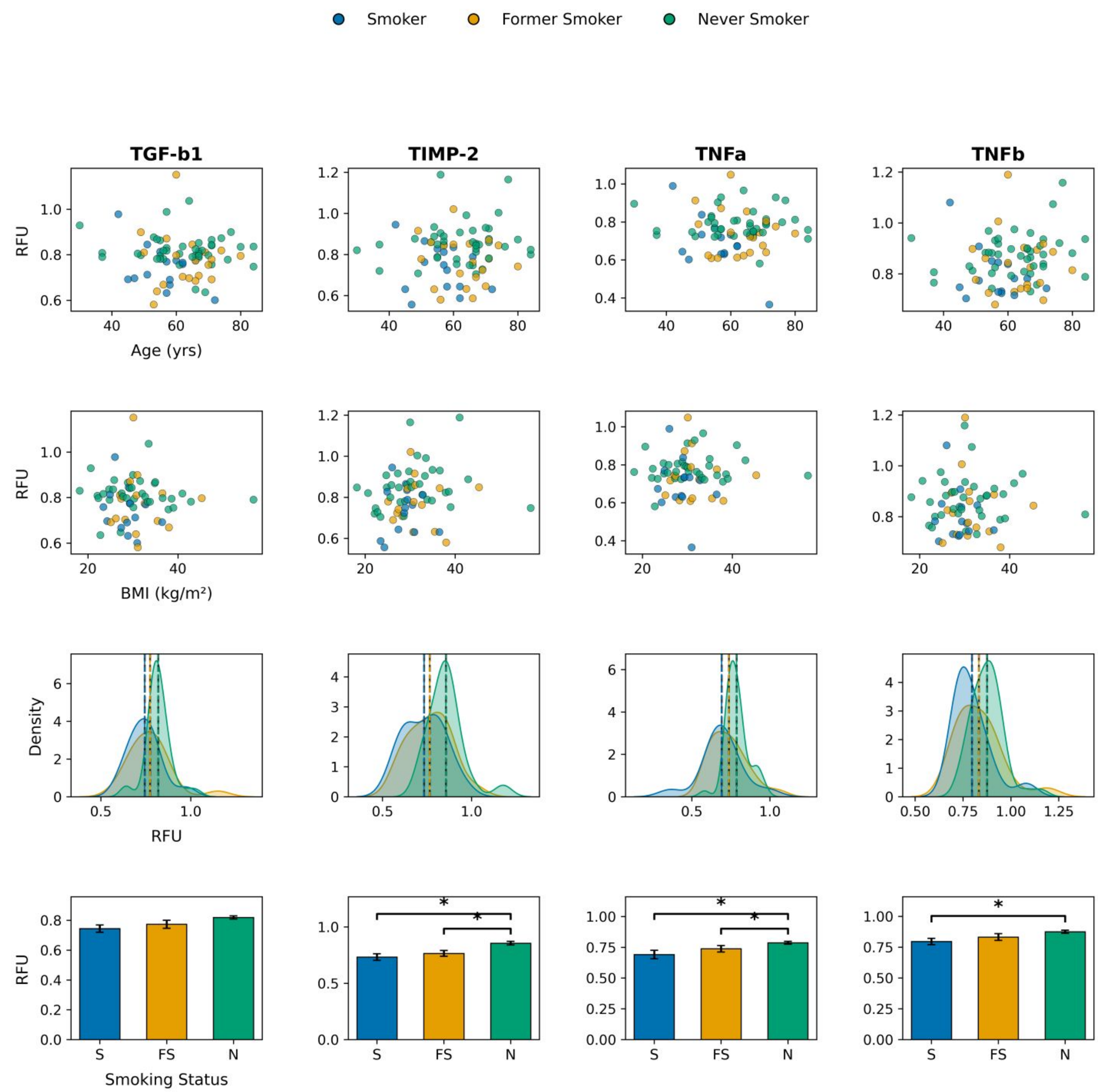

Figure S10. Smoking specific analysis of: TGF-b1, TIMP-2, TNFa, TNFb. *Statistically significant (FDR p<0.05) proteins from Duns Post Hoc analysis: Visualization of the relative normalized fluorescent units (RFU) for each protein (3 of 40). For each protein column (i.e., protein), the subsequent rows depict*

(a) Scatter plot of normalized RFU vs. patient age.
(b) Scatter plot of normalized RFU vs. patient BMI.
(c) Kernel density plot showing distribution of RFU across smoking cohorts, with dashed vertical lines indicating group means, Density plots illustrate the relative distribution and variability of signal intensity within each cohort.
(d) Group comparison of mean normalized fluorescent signal intensity ± SEM across smoking categories. Statistically significant differences between a group are denoted by the asterisk (*).

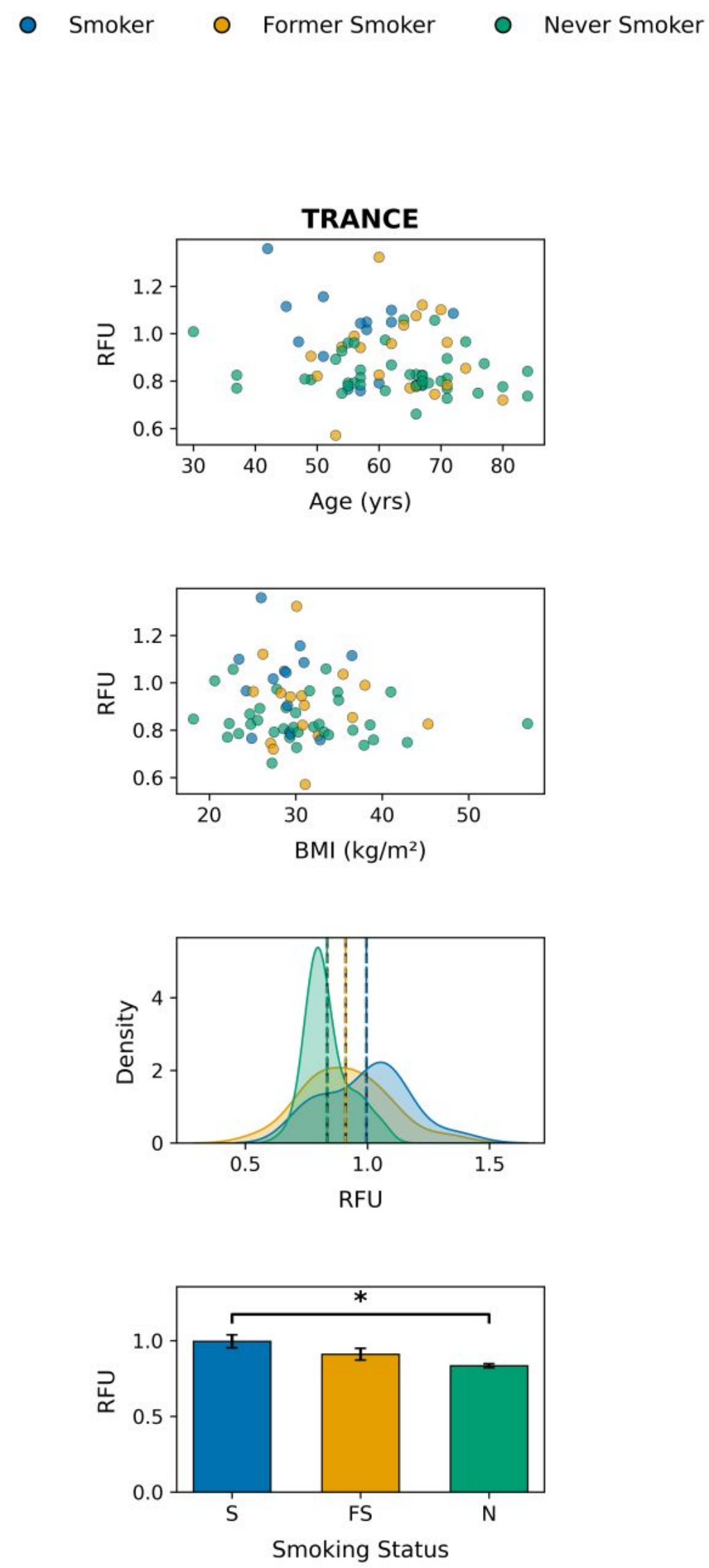

Figure S11. Smoking specific analysis of: TRANCE. *Statistically significant (FDR p<0.05) proteins from Duns Post Hoc analysis: Visualization of the relative normalized fluorescent units (RFU) for each protein (4 of 40). For each protein column (i.e., protein), the subsequent rows depict*

(a) Scatter plot of normalized RFU vs. patient age.

(b) Scatter plot of normalized RFU vs. patient BMI.

(c) Kernel density plot showing distribution of RFU across smoking cohorts, with dashed vertical lines indicating group means, Density plots illustrate the relative distribution and variability of signal intensity within each cohort.

(d) Group comparison of mean normalized fluorescent signal intensity ± SEM across smoking categories. Statistically significant differences between a group are denoted by the asterisk (*).
